# Type 2 Diabetes and Risk of Incident Cancer in China: A Prospective Study Among 0.5 Million Chinese Adults

**DOI:** 10.1093/aje/kwx376

**Published:** 2018-01-03

**Authors:** Xiong-Fei Pan, Meian He, Canqing Yu, Jun Lv, Yu Guo, Zheng Bian, Ling Yang, Yiping Chen, Tangchun Wu, Zhengming Chen, An Pan, Liming Li, China Kadoorie Biobank Collaborative Group

**Affiliations:** 1 Department of Epidemiology and Biostatistics, Ministry of Education Key Laboratory of Environment and Health and State Key Laboratory of Environmental Health; 2 Department of Occupational and Environmental Health, Ministry of Education Key Laboratory of Environment and Health and State Key Laboratory of Environmental Health; 3 Department of Epidemiology and Biostatistics, School of Public Health, Peking University Health Science Center, Beijing, China; 4 Chinese Academy of Medical Sciences, Beijing, China; 5 Clinical Trial Service Unit and Epidemiological Studies Unit, Nuffield Department of Population Health, University of Oxford, Oxford, United Kingdom

**Keywords:** blood glucose concentration, breast cancer, cancer, cohort studies, diabetes mellitus, type 2, liver cancer, pancreatic cancer, risk

## Abstract

Using data from the China Kadoorie Biobank Study, we conducted a prospective investigation on the association between type 2 diabetes mellitus (T2DM) and cancer risk in Chinese adults. A total of 508,892 participants (mean age = 51.5 (standard deviation, 10.7) years) without prior cancer diagnosis at baseline (2004–2008) were included. We documented 17,463 incident cancer cases during follow-up through December 31, 2013. Participants with T2DM had increased risks of total and certain site-specific cancers; hazard ratios were 1.13 (95% confidence interval (CI): 1.07, 1.19) for total cancer, 1.51 (95% CI: 1.29, 1.76) for liver cancer, 1.86 (95% CI: 1.43, 2.41) for pancreatic cancer, and 1.21 (95% CI: 1.01, 1.47) for female breast cancer. The associations were largely consistent when physician-diagnosed and screen-detected T2DM were analyzed separately, except for colorectal cancer (for physician-diagnosed T2DM, HR = 0.91 (95% CI: 0.73, 1.13), and for screen-detected T2DM, HR = 1.44 (95% CI: 1.18, 1.77)). In participants without a prior diagnosis of T2DM, higher random blood glucose levels were positively associated with risks of total cancer, liver cancer, and female breast cancer (all *P*’s for trend ≤ 0.02). In conclusion, T2DM is associated with an increased risk of new-onset cancer in China, particularly cancers of the liver, pancreas, and female breast.

Diabetes and cancer are major public health threats in China. In a recent national survey, Wang et al. (1) reported that 10.9% of Chinese adults had diabetes in 2013, and more importantly, 35.7% had prediabetes. As for cancer, approximately 4.3 million new cancer cases and 2.8 million cancer deaths occurred in China in 2015 alone (2). The burden of diabetes and cancer may continue to rise because of the demographic and social transitions occurring in China, such as urbanization, increasingly sedentary lifestyles, overnutrition, and aging.

The link between diabetes and different cancers has been studied extensively in different populations (3–7). In a recent comprehensive review, Tsilidis et al. (8) assessed the evidence from 27 meta-analyses on the link between type 2 diabetes mellitus (T2DM) and different types of cancer. Although investigators in 20 of the included meta-analyses reported significant results, Tsilidis et al. concluded that there was robust evidence only for the associations between T2DM and breast cancer, intrahepatic cholangiocarcinoma, colorectal cancer, and endometrial cancer (8). Most of the included meta-analyses had substantial heterogeneity that could not be easily explained by the study design, sex composition, or other important risk factors for cancer, and thus some of the reported associations could have been false-positives or inflated. Findings from the review have raised controversy over the association between diabetes and site-specific cancers in different studies or populations.

Meanwhile, no consensus has been reached regarding the association between diabetes and cancer risk in the Chinese population. In a recent pooled-analysis of 19 Asian cohort studies (including 7 in Chinese populations), Chen et al. (9) reported that T2DM was associated with a 26% increased risk of cancer mortality, especially mortality from cancers of the colon and rectum, liver, bile duct, gallbladder, pancreas, breast, endometrium, ovary, prostate, kidney, and thyroid, as well as lymphoma. In another large prospective study using data from the China Kadoorie Biobank (CKB), with over 0.5 million people, Bragg et al. (10) reported that T2DM was associated with an increased risk of mortality from cancers of the liver, pancreas, female breast, and female reproductive system. However, the associations between T2DM and incident cancer were not examined in the pooled analysis or in the CKB Study. Investigators recently reported an increased risk of incident pancreatic cancer associated with diabetes on the basis of the CKB data (11).

Therefore, we comprehensively examined the associations between baseline T2DM and risk of incident cancer in the Chinese population using the CKB data. The association between baseline random blood glucose (RBG) concentration and cancer risk was also assessed among participants without a prior T2DM diagnosis. We assessed such associations for all cancers combined and for major site-specific cancers.

## METHODS

### Study population

Details on the CKB Study design, methods, and procedures have been provided elsewhere (12, 13). Briefly, permanent residents aged 35–74 years from 100–150 rural villages or urban residential committees in 10 diverse regions (5 rural counties and 5 urban districts) across China were invited to participate between June 2004 and July 2008. The CKB Study successfully recruited a total of 512,891 participants aged 30–79 years (41% men, 56% from rural areas; mean age = 52 years), including 12,668 participants slightly outside of the originally designed age range of 35–74 years.

### Baseline data collection

Trained staff interviewed participants at baseline using a standardized electronic questionnaire requesting information on demographic and socioeconomic characteristics, lifestyle and behaviors (such as cigarette smoking, alcohol drinking, diet, and physical activity), general health (such as disease history and current medication use), family history of disease (such as diabetes and cancer), mental disorders, and reproductive history (for women). Physical activity was estimated in terms of metabolic equivalent of task hours per day spent on work, transportation, housework, and nonsedentary recreation. Study staff took anthropometric measurements, such as weight and height, and basic physical measurements, such as blood pressure, using calibrated instruments according to standardized protocols. Ten-milliliter nonfasting venous blood samples (with a record of the time since the participant last ate) were collected from participants using ethylenediaminetetraacetic acid Vacutainers (BD Hemogard; Becton Dickinson, Franklin Lakes, New Jersey). Blood spot tests for measurement of blood glucose level were conducted on-site using a SureStep Plus meter (LifeScan, Inc., Chesterbrook, Pennsylvania) that was regularly calibrated with the manufacturer’s quality control solution. Participants who did not report physician-diagnosed diabetes but had an RBG level of 7.8–11.0 mmol/L were invited to undergo a fasting glucose test the next day at the project site. Prior physician-diagnosed diabetes was defined by a “yes” answer to the question, “Has a physician ever told you that you had diabetes?”. For reported diabetes, persons diagnosed at an age below 30 years and being treated with insulin at baseline enrollment were excluded as probable cases of type 1 diabetes (14). Screen-detected T2DM was defined as no previous physician-diagnosed diabetes but the presence of: 1) an RBG level ≥7.0 mmol/L and a fasting time ≥8 hours; 2) an RBG level ≥11.1 mmol/L and a fasting time <8 hours; or 3) a fasting blood glucose level ≥7.0 mmol/L (10). In this study, the primary exposure of interest was T2DM, which included both prior physician-diagnosed T2DM and screen-detected T2DM.

### Follow-up and endpoint definitions

Study participants were followed up for morbidity and mortality information, mainly through existing disease monitoring systems (12). Mortality information was obtained from the Chinese Center for Disease Control and Prevention’s National Disease Surveillance Points system, checked annually against local residential and medical records and death certificates, and supplemented by active confirmation through street committee or village administrators. Causes of death were coded using the *International Statistical Classification of Diseases and Related Health Problems, Tenth Revision* (ICD-10), by trained staff blinded to baseline information. Morbidity information was collected through linkage with established disease registries for major diseases, such as cancer and diabetes, and the national health insurance system, which records the ICD-10 codes of hospitalizations.

Primary outcomes for the current study were total incident cancers (ICD-10 codes C00–C97), as well as site-specific cancers according to ICD-10 codes. Since there were few incident cases (<50) of some rare types of cancer among participants with T2DM, we focused on several major cancer types, including cancers of the esophagus, stomach, colon and rectum, liver, pancreas, lung, and female breast, which were also the most prevalent cancers in China (2).

### Statistical analysis

We excluded 2,578 participants with a baseline history of physician-diagnosed cancer and 1,340 participants without information on parental history of cancer. In addition, we excluded 2 participants without data on body mass index (weight (kg)/height (m)^2^) and 44 women without data on menopausal status. Thirty-five participants were excluded because they were judged highly likely to be type 1 diabetes cases. Finally, a total of 508,892 persons (208,832 men (41.0%)) were included for the main analyses.

Person-years were calculated as duration of time from baseline enrollment to the onset of cancer, death, loss to follow-up, or December 31, 2013, whichever came first. Cox proportional hazards regression models were used to analyze associations and generate hazard ratios and 95% confidence intervals. The proportional hazards assumption was tested by dividing the follow-up period into 3 intervals that held similar numbers of incident cancer cases and comparing effect sizes of T2DM for risk of incident cancer; no evidence of departure from the assumption was found. Incidence rates of cancers were estimated through direct standardization by sex, age (5-year age intervals), and study area, with the total study population as the standard.

We stratified the analysis by sex, age (5-year intervals), and study area in the first model, and additionally controlled for educational level, parental history of cancer, and 4 modifiable risk factors (body mass index as a continuous variable, cigarette smoking, alcohol drinking, and physical activity) in the second model. Menopausal status was included for association analyses for female breast cancer. Data on all variables were taken from the baseline surveys. Adjusted hazard ratios were also calculated across strata of other risk factors (sex, age, educational level, household registration, parental history of cancer, body mass index, cigarette smoking, alcohol drinking, physical activity, and menopause), and χ^2^ tests for trend or heterogeneity were applied to the log hazard ratios and their standard errors (11, 15).

Moreover, we conducted separate analyses for physician-diagnosed T2DM and screen-detected T2DM, with a common reference group of participants without T2DM. We also conducted sensitivity analyses by 1) excluding participants who had cancer diagnosed, died, or were lost to follow-up within the first 3 years of follow-up; 2) treating T2DM as a time-varying variable during follow-up (i.e., the status of T2DM was time-updated, but diabetes information during follow-up was obtained solely from the disease surveillance systems); 3) excluding persons with major prior noncancer diseases (coronary heart disease, rheumatic heart disease, stroke, transient ischemic attack, and hepatitis/cirrhosis); and 4) additionally adjusting for frequency of consumption of fresh fruit, vegetables, and meat in the model. The association between time since T2DM diagnosis (from the first diagnosis date to the baseline) and the risk of incident cancer was assessed after excluding screen-detected T2DM. In addition, we analyzed the association between baseline RBG and risk of incident cancer among participants without a history of physician-diagnosed T2DM. Because the association between diabetes and pancreatic cancer has already been reported in the CKB Study (11), we did not repeatedly conduct subgroup or sensitivity analyses for pancreatic cancer.

All analyses were conducted using Stata 14 (StataCorp LP, College Station, Texas). All *P* values were 2-sided, and statistical significance was defined as *P* < 0.05.

## RESULTS

### Characteristics of study participants

Among the 508,892 study participants without a prior cancer diagnosis, the mean age at baseline was 51.5 (standard deviation, 10.7) years. A total of 29,835 participants (5.9%) had T2DM at baseline, of whom 15,881 (3.1% of all participants) reported physician-diagnosed T2DM and 13,954 (2.7% of all participants) had screen-detected T2DM (Table 1). Compared with participants without diabetes, those who had T2DM were older, more likely to be female and urban residents, had higher body mass indices, and were more likely to be postmenopausal (if female), former regular smokers or alcohol drinkers, and less physically active. Educational level and parental history of cancer also differed significantly among participants with and without T2DM. Among 15,881 participants who reported prior physician-diagnosed T2DM, the median age at diabetes diagnosis was 53 years (interquartile range, 47–60 years), and the median time since diagnosis was 4 years (interquartile range, 2–8 years).

**Table 1.** Baseline Characteristics of Participants According to Type 2 Diabetes Mellitus Status, China Kadoorie Biobank Study, 2004–2008

Characteristic	T2DM Status						*P* Value^a^
	Total		No T2DM		T2DM		
	No. of Persons	%	No. of Persons	%	No. of Persons	%	
Total	508,892	100.0	479,057	94.1	29,835	5.9	
Sex							<0.001
Male	208,832	41.0	197,302	41.2	11,530	38.6	
Female	300,060	59.0	281,755	58.8	18,305	61.4	
Age group, years							<0.001
30–59	385,861	75.8	369,070	77.0	16,791	56.3	
60–69	90,597	17.8	81,237	17.0	9,360	31.4	
70–79	32,434	6.4	28,750	6.0	3,684	12.3	
Educational level							<0.001
Primary or illiterate	258,151	50.7	242,182	50.5	15,969	53.5	
Above primary	250,741	49.3	236,875	49.5	13,866	46.5	
Household registration							<0.001
Rural	285,033	56.0	273,291	57.1	11,742	39.4	
Urban	223,859	44.0	205,766	42.9	18,093	60.6	
Menopausal status^b^							<0.001
Premenopausal	128,443	42.8	125,441	44.5	3,002	16.4	
Perimenopausal	14,733	4.9	14,002	5.0	731	4.0	
Postmenopausal	156,884	52.3	142,312	50.5	14,572	79.6	
Body mass index^c^							<0.001
Underweight (<18.5)	22,076	4.3	21,363	4.5	713	2.4	
Normal (18.5–23.9)	264,063	51.9	253,220	52.8	10,843	36.3	
Overweight (24.0–27.9)	168,876	33.2	156,440	32.7	12,436	41.7	
Obese (≥28.0)	53,877	10.6	48,034	10.0	5,843	19.6	
Cigarette smoking							<0.001
Never smoker	314,968	61.9	295,886	61.8	19,082	64.0	
Occasional smoker	28,958	5.7	27,404	5.7	1,554	5.2	
Former regular smoker	30,029	5.9	27,246	5.7	2,783	9.3	
Current smoker	134,937	26.5	128,521	26.8	6,416	21.5	
Alcohol drinking							<0.001
Never regular drinker	233,079	45.8	217,424	45.4	15,655	52.5	
Former regular drinker	9,041	1.8	8,128	1.7	913	3.1	
Occasional drinker	179,379	35.2	170,867	35.7	8,512	28.5	
Weekly drinker	87,393	17.2	82,638	17.2	4,755	15.9	
Physical activity^d^, MET-hours/day							<0.001
<10.0	119,816	23.5	107,894	22.5	11,922	40.0	
10.0–14.9	94,602	18.6	87,825	18.3	6,777	22.7	
≥15.0	294,474	57.9	283,338	59.2	11,136	37.3	
Parental history of cancer							<0.01
No	437,710	86.0	412,218	86.0	25,492	85.4	
Yes	71,182	14.0	66,839	14.0	4,343	14.6	

Abbreviations: MET, metabolic equivalent of task; T2DM, type 2 diabetes mellitus.

^a^*P* values were calculated by *t* test for continuous variables and χ^2^ test for categorical variables.

^b^ For women only (*n* = 300,060).

^c^ Weight (kg)/height (m)^2^.

^d^ Physical activity was estimated in terms of MET-hours/day spent on work, transportation, housework, and nonsedentary recreation.

### Association between T2DM and cancer risk

**Figure 1.**
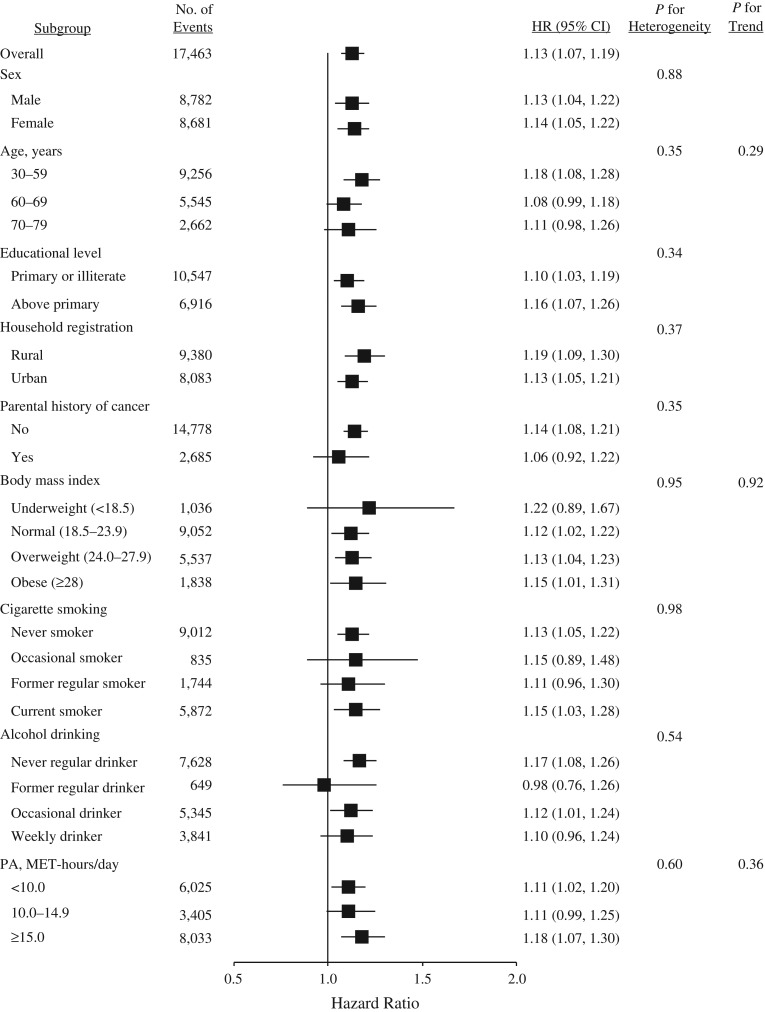
Adjusted hazard ratios (HRs) for all cancers combined according to type 2 diabetes mellitus status, China Kadoorie Biobank Study, 2004–2013. Body mass index was defined as weight (kg)/height (m)^2^; physical activity (PA) was estimated in terms of MET-hours/day spent on work, transportation, housework, and nonsedentary recreation. Bars, 95% confidence intervals (CIs). MET, metabolic equivalent of task.

**Figure 2.**
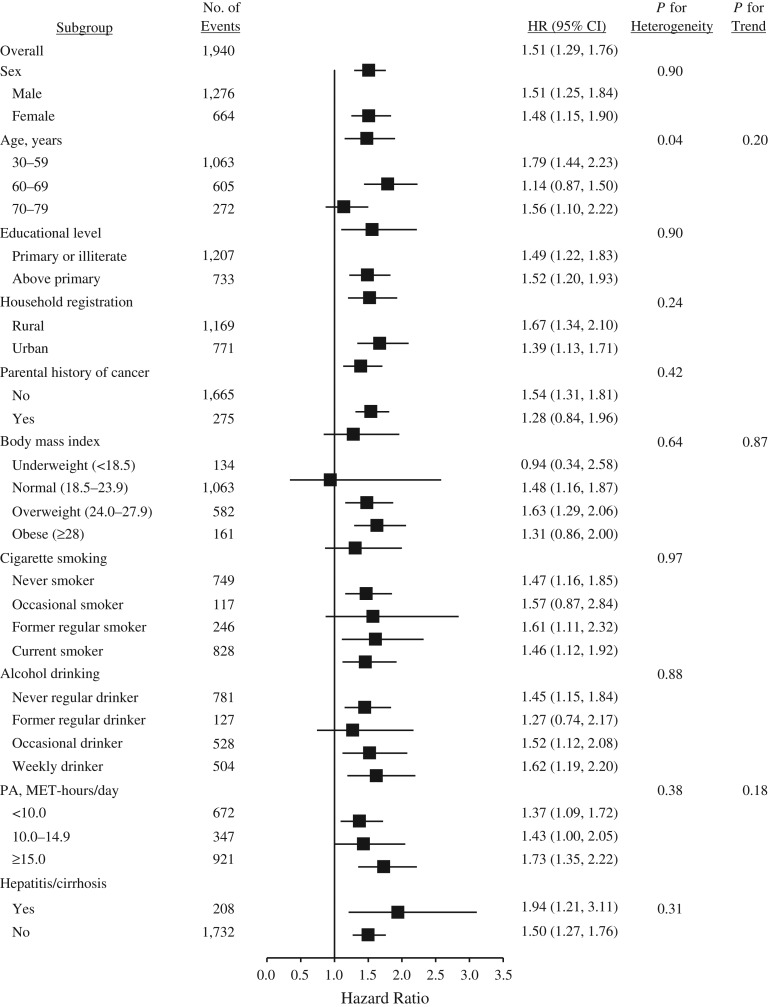
Adjusted hazard ratios (HRs) for liver cancer according to type 2 diabetes mellitus status, China Kadoorie Biobank Study, 2004–2013. Body mass index was defined as weight (kg)/height (m)^2^; physical activity (PA) was estimated in terms of MET-hours/day spent on work, transportation, housework, and nonsedentary recreation. Bars, 95% confidence intervals (CIs). MET, metabolic equivalent of task.

**Figure 3.**
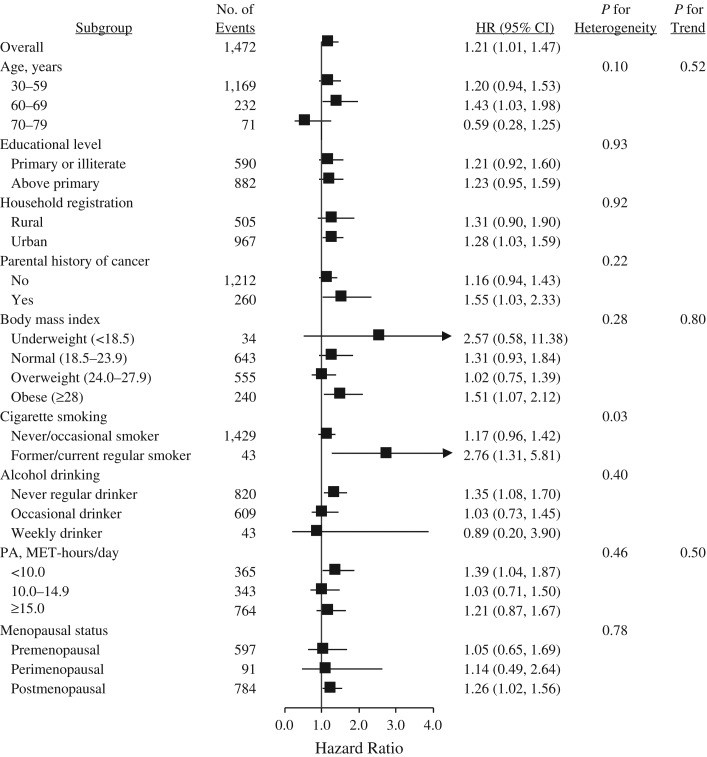
Adjusted hazard ratios (HRs) for female breast cancer according to type 2 diabetes mellitus status, China Kadoorie Biobank Study, 2004–2013. Body mass index was defined as weight (kg)/height (m)^2^; physical activity (PA) was estimated in terms of MET-hours/day spent on work, transportation, housework, and nonsedentary recreation. Bars, 95% confidence intervals (CIs). MET, metabolic equivalent of task.

A total of 17,463 participants developed cancer during 3,612,769 person-years of follow-up (mean follow-up = 7.1 years), including 1,457 cancers among participants with T2DM and 16,006 among those without T2DM (Table 2). Overall, participants with T2DM at baseline were 1.13 times as likely to develop cancer (hazard ratio (HR) = 1.13, 95% confidence interval (CI): 1.07, 1.19). With respect to site-specific cancers, hazard ratios were 1.51 (95% CI: 1.29, 1.76) for liver cancer, 1.86 (95% CI: 1.43, 2.41) for pancreatic cancer, and 1.21 (95% CI: 1.01, 1.47) for female breast cancer. In addition, no heterogeneity was observed for the associations between T2DM and overall or site-specific cancers by sex, educational level, household registration, parental history of cancer, body mass index, alcohol drinking, physical activity, and menopause (if female) (Figures 1–3; also see Web Figures 1–4, available at https://academic.oup.com/aje), although associations of T2DM with liver cancer and female breast cancer differed statistically as regards age and cigarette smoking, respectively (Figures 2 and 3). The association with liver cancer was higher in rural residents (HR = 1.67, 95% CI: 1.34, 2.10) than in urban residents (HR = 1.39, 95% CI: 1.13, 1.71), and higher among persons with hepatitis/cirrhosis (HR = 1.94, 95% CI: 1.21, 3.11) than among those without it (HR = 1.50, 95% CI: 1.27, 1.76), despite no statistical heterogeneity (Figure 2). The positive association with female breast cancer was observed in postmenopausal women (HR = 1.26, 95% CI: 1.02, 1.56) but not in perimenopausal (HR = 1.14, 95% CI: 0.49, 2.64) or premenopausal (HR = 1.05, 95% CI: 0.65, 1.69) women (Figure 3).

**Table 2.** Association Between Type 2 Diabetes Mellitus and Risk of Incident Cancer in the China Kadoorie Biobank Study, 2004–2013

Type of Cancer	T2DM Status				Risk of Cancer in Persons With Diabetes (Relative to No Diabetes)^a^			
	T2DM		No T2DM					
	No. of Cases	Rate per 100,000 P-Y^b^	No. of Cases	Rate per 100,000 P-Y^b^	Model 1^c^		Model 2^d^	
					HR	95% CI	HR	95% CI
All cancers	1,457	576.3	16,006	491.7	1.12	1.06, 1.18	1.13	1.07, 1.19
Esophagus	87	40.8	1,572	48.2	0.79	0.64, 0.99	0.86	0.69, 1.08
Stomach	148	56.5	2,061	63.4	0.88	0.74, 1.04	0.91	0.77, 1.08
Colon and rectum	190	63.1	1,721	53.1	1.16	0.99, 1.35	1.13	0.97, 1.32
Liver	194	86.1	1,746	52.8	1.50	1.29, 1.74	1.51	1.29, 1.76
Pancreas	71	23.5	427	13.1	1.83	1.42, 2.37	1.86	1.43, 2.41
Lung	310	107.2	3,217	99.7	1.05	0.93, 1.18	1.11	0.98, 1.25
Female breast^e^	128	79.1	1,344	67.2	1.24	1.03, 1.50	1.21	1.01, 1.47

Abbreviations: CI, confidence interval; HR, hazard ratio; P-Y, person-years; T2DM, type 2 diabetes mellitus.

^a^ T2DM was treated as a fixed baseline variable for analyses.

^b^ Standardized to the sex, age (5-year intervals), and study area of the study population.

^c^ Model 1 stratified by sex, age (5-year intervals), and study area of the study population.

^d^ Model 2 stratified by sex, age (5-year intervals), and study area of the study population. Results were adjusted for education, parental history of cancer, body mass index, cigarette smoking, alcohol drinking, and physical activity.

^e^ For women only (*n* = 300,060). Rates were standardized to the age and study area of the study population. Model 2 additionally adjusted for education, parental history of cancer, body mass index, cigarette smoking, alcohol drinking, physical activity, and menopausal status.

The associations between T2DM and risk of incident cancer were largely consistent when physician-diagnosed and screen-detected T2DM were analyzed separately, except for colorectal cancer (Table 3). The association with colorectal cancer was positive among persons with screen-detected T2DM but was insignificantly negative among persons with physician-diagnosed T2DM. The associations between T2DM and risk of incident cancer remained materially unchanged in sensitivity analyses after exclusion of new-onset cancers, deaths, and losses to follow-up occurring within the first 3 years of follow-up (Web Table 1), after excluding persons with major prior noncancer diseases at baseline (Web Table 2), and in the model with additional adjustment for consumption of fresh fruit, vegetables, and meat (as well as hepatitis/cirrhosis for liver cancer) (Web Table 3). Although the association between T2DM and lung cancer became statistically significant when diagnosed diabetes was treated as time-varying in the sensitivity analysis, the associations for other cancer types were consistent with results in the main analyses (Web Table 4).

**Table 3.** Association Between Type 2 Diabetes Mellitus and Risk of Incident Cancer, by Method of Diabetes Detection, China Kadoorie Biobank Study, 2004–2013

Type of Cancer	No. of Events Among Persons Without T2DM	Method of T2DM Detection						*P* Value^a^
		Screen-Detected T2DM			Physician-Diagnosed T2DM			
		No. of Events	HR^b^	95% CI	No. of Events	HR^b^	95% CI	
All cancers	16,006	653	1.17	1.08, 1.27	804	1.10	1.02, 1.18	0.20
Esophagus	1,572	49	1.01	0.76, 1.35	38	0.72	0.52, 1.00	0.12
Stomach	2,061	76	1.06	0.84, 1.33	72	0.79	0.63, 1.01	0.08
Colon and rectum	1,721	100	1.44	1.18, 1.77	90	0.91	0.73, 1.13	0.001
Liver	1,746	94	1.67	1.35, 2.06	100	1.37	1.12, 1.69	0.18
Lung	3,217	129	1.11	0.93, 1.33	181	1.11	0.95, 1.29	0.96
Female breast^c^	1,344	55	1.19	0.91, 1.56	73	1.23	0.97, 1.57	0.85

Abbreviations: CI, confidence interval; HR, hazard ratio; T2DM, type 2 diabetes mellitus.

^a^*P* values were calculated in a Wald test comparing the risk of incident cancer in relation to screen-detected diabetes with the risk in relation to physician-diagnosed diabetes.

^b^ The model stratified by the sex, age (5-year intervals), and study area of the study population. Results were adjusted for education, parental history of cancer, body mass index, cigarette smoking, alcohol drinking, and physical activity.

^c^ For women only (*n* = 300,060). The model stratified by the age (5-year intervals) and study area of the study population. Results were adjusted for education, parental history of cancer, menopausal status, body mass index, cigarette smoking, alcohol drinking, and physical activity.

Among the participants with a diagnosis of T2DM, the association with liver cancer was stronger in those whose T2DM was diagnosed less than or equal to 5 years prior to baseline (HR = 1.56, 95% CI: 1.20, 2.04) than in those whose T2DM was diagnosed over 5 years prior to diagnosis (HR = 1.18, 95% CI: 0.87, 1.61; Table 4). However, associations were stronger with all cancers combined (*P* for trend = 0.02) and female breast cancer (*P* for trend = 0.02) among participants with longer time from diabetes diagnosis to baseline.

**Table 4.** Association Between Type 2 Diabetes Mellitus and Risk of Incident Cancer According to Time From Diabetes Diagnosis to Baseline, China Kadoorie Biobank Study, 2004–2013^a^

Type of Cancer	No. of Events Among Persons Without T2DM	Time From T2DM Diagnosis to Baseline						*P* for Trend^b^
		≤5 Years			>5 Years			
		No. of Events	HR^c^	95% CI	No. of Events	HR^c^	95% CI	
All cancers	16,006	391	1.09	0.99, 1.21	413	1.10	0.99, 1.21	0.02
Esophagus	1,572	19	0.71	0.45, 1.11	19	0.73	0.46, 1.17	0.06
Stomach	2,061	28	0.63	0.44, 0.92	44	0.96	0.71, 1.30	0.20
Colon and rectum	1,721	41	0.87	0.64, 1.19	49	0.94	0.70, 1.25	0.44
Liver	1,746	57	1.56	1.20, 2.04	43	1.18	0.87, 1.61	0.02
Lung	3,217	83	1.08	0.86, 1.34	98	1.12	0.91, 1.37	0.23
Female breast^d^	1,344	28	0.94	0.64, 1.38	45	1.56	1.15, 2.12	0.02

Abbreviations: CI, confidence interval; HR, hazard ratio; T2DM, type 2 diabetes mellitus.

^a^ Participants with screen-detected T2DM (*n* = 13,954) and an implausible age of diagnosis (*n* = 20) were excluded from the analysis, and the total sample size was 494,918.

^b^*P* values for trend were from a likelihood ratio test comparing the model with time since diabetes diagnosis as an ordered categorical variable to the model without it.

^c^ The model stratified by the sex, age (5-year intervals), and study area of the study population. Results were adjusted for education, parental history of cancer, body mass index, cigarette smoking, alcohol drinking, and physical activity.

^d^ For women only (*n* = 291,587). The model stratified by the age (5-year intervals) and study area of the study population. Results were adjusted for education, parental history of cancer, menopausal status, body mass index, cigarette smoking, alcohol drinking, and physical activity.

In persons without a prior diagnosis of T2DM, there was an increasing trend in the associations between RBG level and risk of all cancers combined, stomach cancer, colorectal cancer, liver cancer, and female breast cancer (*P*’s for trend ≤0.02 for all; Table 5).

**Table 5.** Association Between Random Blood Glucose Level and Risk of Incident Cancer Among Participants Without Prior Physician-Diagnosed Type 2 Diabetes, China Kadoorie Biobank Study, 2004–2013^a^

Type of Cancer	Random Blood Glucose Level							*P* for Trend^b^
	≤5.5 mmol/L	5.6–6.9 mmol/L			≥7.0 mmol/L			
	(No. of Events)	No. of Events	HR^c^	95% CI	No. of Events	HR^c^	95% CI	
All cancers	7,263	6,020	1.07	1.04, 1.11	3,057	1.20	1.15, 1.25	<0.001
Esophagus	712	577	1.14	1.02, 1.27	299	1.15	1.00, 1.31	0.56
Stomach	918	763	1.08	0.98, 1.20	423	1.25	1.11, 1.41	0.01
Colon and rectum	757	664	1.05	0.95, 1.17	366	1.23	1.08, 1.40	<0.01
Liver	789	629	1.11	0.99, 1.23	379	1.44	1.27, 1.63	<0.001
Lung	1,478	1,211	1.06	0.98, 1.14	593	1.11	1.01, 1.22	0.08
Female breast^d^	606	524	1.05	0.93, 1.18	253	1.30	1.11, 1.51	0.02

Abbreviations: CI, confidence interval: HR, hazard ratio.

^a^ Participants with prior physician-diagnosed diabetes (*n* = 15,881) and no data on random blood glucose level (*n* = 8,111) were excluded from the analysis, and the total sample size was 484,900.

^b^*P* values for trend were from a likelihood ratio test comparing the model with random blood glucose as a continuous variable to the model without random blood glucose.

^c^ The model stratified by the sex, age (5-year intervals), and study area of the study population. Results were adjusted for education, parental history of cancer, body mass index, cigarette smoking, alcohol drinking, and physical activity.

^d^ For women only (*n* = 285,448). The model stratified by the age (5-year intervals) and study area of the study population. Results were adjusted for education, parental history of cancer, menopausal status, body mass index, cigarette smoking, alcohol drinking, and physical activity.

## DISCUSSION

This large prospective study in mainland China showed that persons with T2DM had significantly increased risk of developing cancer (mainly cancers of the liver, pancreas, and female breast) compared with persons without T2DM. The findings were consistent with those from a report by the American Diabetes Association and the American Cancer Society (16), as well as from an earlier study on the association between diabetes and cancer mortality using the CKB data (10). We also found that positive associations with incident cancer existed for both physician-diagnosed and screen-detected T2DM. In addition, higher RBG level was associated with a linearly increased risk of total cancer and cancers of the liver and female breast.

Our study found that T2DM was associated with a 13% increased risk of total cancer, which was slightly lower than the results from a recent pooled analysis of 19 prospective cohort studies in Asians (for the relationship between diabetes and cancer mortality, HR = 1.26) (9). However, whether this was due to different outcome measures was unclear, because in a previous meta-analysis of studies conducted mainly in Western populations, Noto et al. (17) also reported a minor difference in effect sizes (relative risk = 1.17 for incident cancer vs. 1.21 for cancer mortality). Overall, our findings and others demonstrate a robust and reliable link between diabetes and cancer in the Chinese population.

The pooled analysis of Asian cohort studies showed an approximately 2-fold increased risk of mortality from liver cancer in persons with diabetes (9). Our estimate (HR = 1.50) was lower than the summary relative risk but was comparable to that found (HR = 1.54) in an earlier CKB study on the association between diabetes and liver cancer mortality (10). Hepatitis infections and nonalcoholic liver diseases were found to be associated with diabetes in earlier studies (18) and could thus potentially confound the association between diabetes and liver cancer. In addition, in their recent review, Wang et al. (19) reported different excess risks of liver cancer associated with T2DM in subgroups by hepatitis status and liver diseases. Such heterogeneity was reflected in 2 studies in Taiwan Chinese that showed positive associations only in persons who were hepatitis C virus–negative (20) and in persons who were both hepatitis B virus– and hepatitis C virus–negative or were positive for antibodies to hepatitis C virus (21). Since hepatitis infections such as hepatitis B and nonalcoholic liver diseases are prevalent in mainland China (22), we tried to delineate the influence of confounding and effect modification of liver diseases on the association between T2DM and diabetes. However, we did not find substantial change in the effect size when we additionally adjusted for liver diseases in the association analysis, or statistical heterogeneity in the subgroup analysis by hepatitis/cirrhosis status.

Our study showed a positive association between T2DM and risk of female breast cancer, which is consistent with the summary relative risk from a meta-analysis of 39 observational studies conducted primarily in Western countries (6). The effect size for incidence of female breast cancer in our study was much lower (1.21 vs. 1.84) than that reported for mortality from female breast cancer in the same cohort (10), which reflects the possibility that comorbidity between T2DM and breast cancer may substantially increase the fatality of breast cancer, although it is generally a malignancy with a good prognosis. In addition, our study found that the hazard ratio point estimate associated with T2DM was higher in postmenopausal women (HR = 1.26) than in perimenopausal (HR = 1.14) or premenopausal (HR = 1.05) women, which is consistent with findings from the meta-analysis that increased risk of female breast cancer was observed among postmenopausal women but not premenopausal women (6). It is suspected that factors such as obesity and changes in concentrations of insulin-like growth factor 1 and estrogens, which contribute to the diabetic state but have differential roles in the etiology of premenopausal and postmenopausal breast cancer, may partly account for the difference in the association (23). Breast cancer has heterogeneous molecular subtypes that are pathologically and prognostically distinct, and hormone receptors such as estrogen receptor may play a role in breast carcinogenesis (24). In the United States, researchers in the Nurses’ Health Study found that associations with diabetes differed substantially among women with estrogen-receptor–positive, estrogen-receptor–negative, and fatal breast cancer (25). Future studies may also need to separately analyze the associations with breast cancer subtypes among Chinese women.

In our study, higher RBG levels were associated with increased risk of total cancer and cancers of stomach, colon and rectum, liver, and female breast among persons without prior physician-diagnosed T2DM. Some of the positive associations existed even in the normal range of blood glucose values, which was consistent with findings from a prospective cohort study in South Korea (26). Besides, a high RBG level (i.e., ≥7.0 mmol/L) rendered risk of almost all other cancer types increased. In contrast, it seemed that a physician’s diagnosis of T2DM less than or equal to 5 years prior to baseline was associated with decreased risk of some cancers, such as stomach cancer. The contrasting findings might imply that diabetes treatments and/or lifestyle changes initiated after a physician’s diagnosis of diabetes potentially decreased the risk of cancer to some extent. Consistently, 2 systematic reviews showed that metformin reduced subsequent cancer risk after diabetes diagnosis (27, 28). However, the evidence of reduced cancer risk associated with different T2DM treatments (e.g., metformin and sulfonylureas) is still inconclusive (29, 30), and large controlled trials are still needed to test the association in the future. Notably, our analysis of time since T2DM was based on diagnosis information recorded at baseline, and this information was not updated during follow-up. Thus, results do not directly correspond to duration of diagnosed T2DM.

The study had major strengths, including its prospective nature, a large sample size, geographic diversity, completeness of data collection, stringent case ascertainment and follow-up mechanisms, and a high retention rate. The findings are thus reliable and generalizable to the general population in China. However, there are certain limitations that need to be addressed. First, since new T2DM cases (previously undiagnosed diabetes) found at baseline were detected only on the basis of RBG level, case misclassification might have been possible. This was partially reflected by the discrepancies between the prevalence of newly diagnosed T2DM in our study (2.7%) and counterpart estimates based on several glycemic indicators, such as fasting plasma glucose, 2-hour plasma glucose, and glycated hemoglobin, in 3 nationally representative studies (5.2% in 2007–2008, 8.1% in 2010, and 6.9% in 2013) (1, 31, 32). Second, the present study was not able to examine the association between T2DM and less common cancers, such as cancers of the prostate, bladder, cervix, and endometrium, that are thought to be associated with diabetes (8, 16). Third, a lack of detailed information about diabetes severity and cancer staging hindered us from estimating the association between severity of T2DM and the onset of cancer to provide additional support for our findings. Fourth, because our information on the exact molecular or pathological types of cancers was inadequate, we could not examine the associations between T2DM and the exact subtypes of certain cancers (e.g., esophageal cancer and breast cancer). Fifth, screening can potentially influence the detection of certain cancer types, such as cancers of the cervix, esophagus, female breast, and colon and rectum. The differential use of cancer screening may confound the association between diabetes and cancer (33). We did not have information on cancer screening for this cohort of Chinese and thus could not control for its impact. However, it is unlikely that people with and without diabetes chose different cancer screening approaches in China during the follow-up period.

In conclusion, our study provides evidence of associations between T2DM and incident cancer, particularly cancers of the liver, pancreas, and female breast, among Chinese adults. Diabetes is undiagnosed among many Chinese adults, and many diagnosed cases are not properly treated (31). Since both diabetes and cancer contribute substantially to the burden of disease in China, our findings, together with an earlier finding based on CKB data that diabetes was positively associated with mortality from similar cancer types (10), demonstrate that close monitoring of T2DM patients for early onset-cancers might be a feasible strategy for cancer prevention. However, such a practice still requires evidence from large clinical trials, which could be a future research direction in this area.

## Supplementary Material

Web Material

## Abbreviations

CI: confidence interval
CKB: China Kadoorie Biobank
HR: hazard ratio
ICD-10: *International Statistical Classification of Diseases and Related Health Problems, Tenth Revision*
RBG: random blood glucose
T2DM: type 2 diabetes mellitus
