# Supplementary material for: Type 2 Diabetes and Risk of Incident Cancer in China: A Prospective Study Among 0.5 Million Chinese Adults
Source: Am J Epidemiol. 2018 Jan 3;187(7):1380–91. doi: 10.1093/aje/kwx376 (PMC6153481; doi:10.1093/aje/kwx376)
Supplement: Web Material [file kwx376panwebmaterialrevised.pdf]

Type 2 Diabetes and Risk of Incident Cancer in China: A Prospective Study among 0.5 Million Chinese Adults

Xiong-Fei Pan, Meian He, Canqing Yu, Jun Lv, Yu Guo, Zheng Bian, Ling Yang, Yiping Chen, Tangchun Wu, Zhengming Chen, An Pan*, and Liming Li*, on behalf of the China Kadoorie Biobank Collaborative Group

* Correspondence to Dr. An Pan, School of Public Health, Tongji Medical College, Huazhong University of Science and Technology, 13 Hangkong Road, Wuhan 430030, China (e-mail: panan@hust.edu.cn); or Dr. Liming Li, School of Public Health, Peking University Health Science Center, 38 Xueyuan Road, Beijing 100191, China (e-mail: lmleeph@vip.163.com).

WEB MATERIAL

Web Figure 1. Adjusted hazard ratios (HRs) for esophageal cancer by type 2 diabetes, China Kadoorie Biobank Study, 2004-2013.

Web Figure 2. Adjusted hazard ratios (HRs) for stomach cancer by type 2 diabetes, China Kadoorie Biobank Study, 2004-2013.

Web Figure 3. Adjusted hazard ratios (HRs) for colorectal cancer by type 2 diabetes, China Kadoorie Biobank Study, 2004-2013.

Web Figure 4. Adjusted hazard ratios (HRs) for lung cancer by type 2 diabetes, China Kadoorie Biobank Study, 2004-2013.

Web Table 1. Association Between Type 2 Diabetes and Risk of Cancer, China Kadoorie Biobank Study, 2004-2013: Excluding Cancer Cases, Deaths, and Loss to Follow-up Occurring Within the First Three Years of Follow-Up

Web Table 2. Association Between Type 2 Diabetes and Risk of Incident Cancer, China Kadoorie Biobank Study, 2004-2013: Excluding Participants With Cardiovascular Disease and Other Major Chronic Disease

Web Table 3. Association Between Type 2 Diabetes and Risk of Incident Cancer, China Kadoorie Biobank Study, 2004-2013: Additional Adjustment for Dietary Factors

Web Table 4. Association Between Type 2 Diabetes and Risk of Cancer, China Kadoorie Biobank Study, 2004-2013: Treating Diagnosed Diabetes Cases as Time-varying During Follow-up

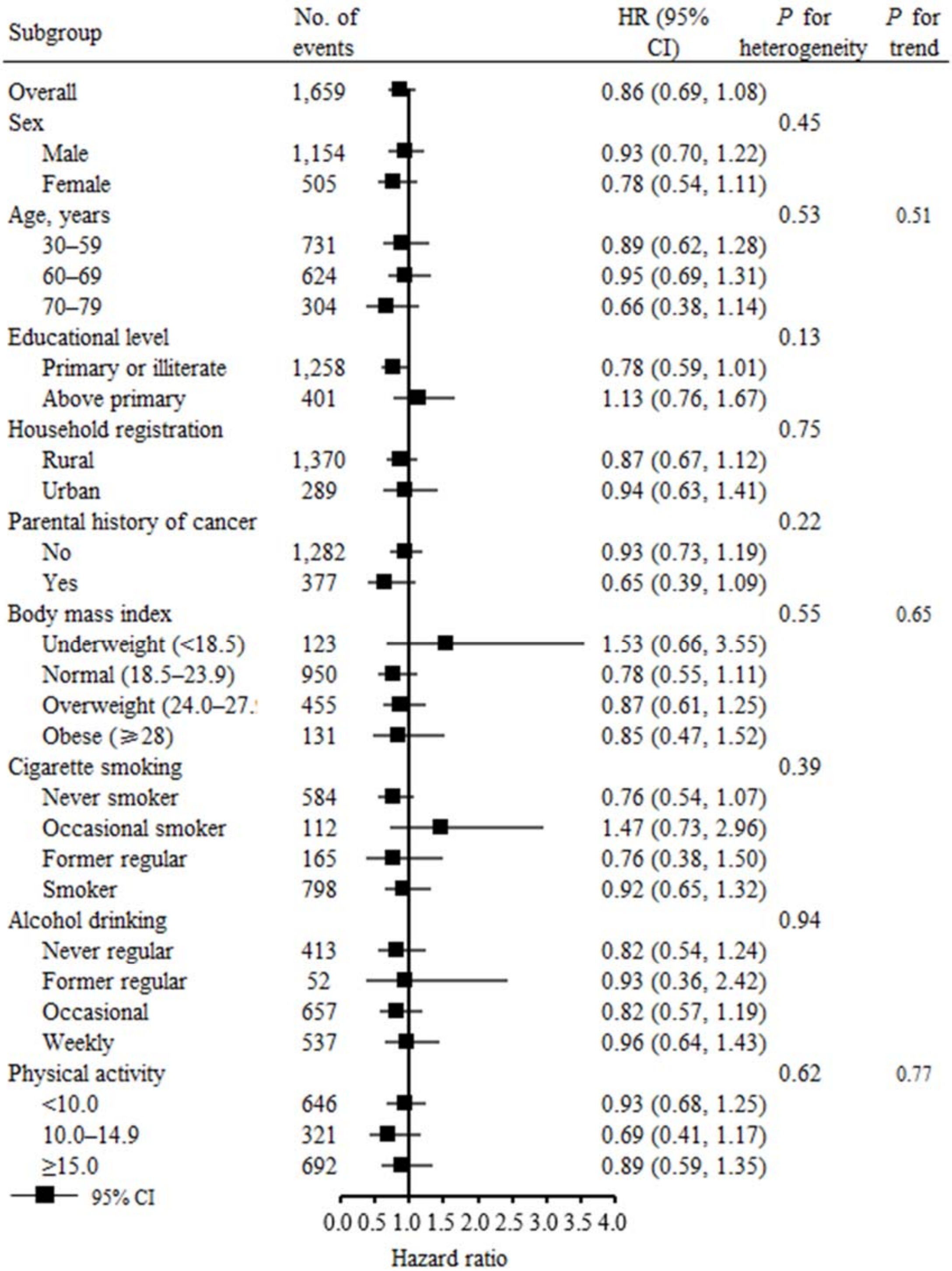

Web Figure 1. Adjusted hazard ratios (HRs) for esophageal cancer by type 2 diabetes, China Kadoorie Biobank Study, 2004-2013. Body mass index was defined as weight (kg)/height (m)^2; physical activity (PA) was estimated in terms of MET-hours/day spent on work, transportation, housework, and nonsedentary recreation. Bars, 95% confidence intervals (CIs). MET, metabolic equivalent of task.

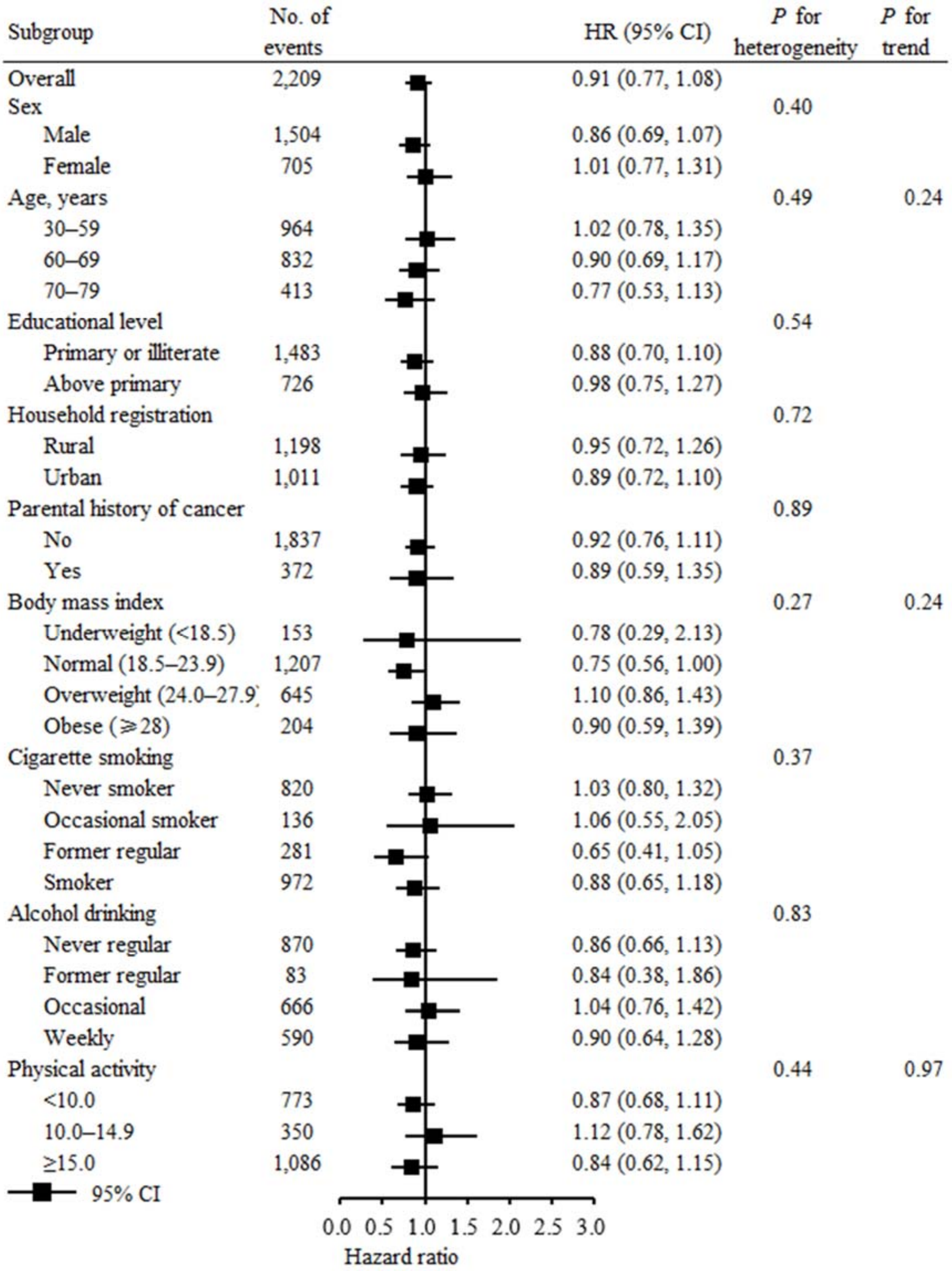

Web Figure 2. Adjusted hazard ratios (HRs) for stomach cancer by type 2 diabetes, China Kadoorie Biobank Study, 2004-2013. Body mass index was defined as weight (kg)/height (m)^2; physical activity (PA) was estimated in terms of MET-hours/day spent on work, transportation, housework, and nonsedentary recreation. Bars, 95% confidence intervals (CIs). MET, metabolic equivalent of task.

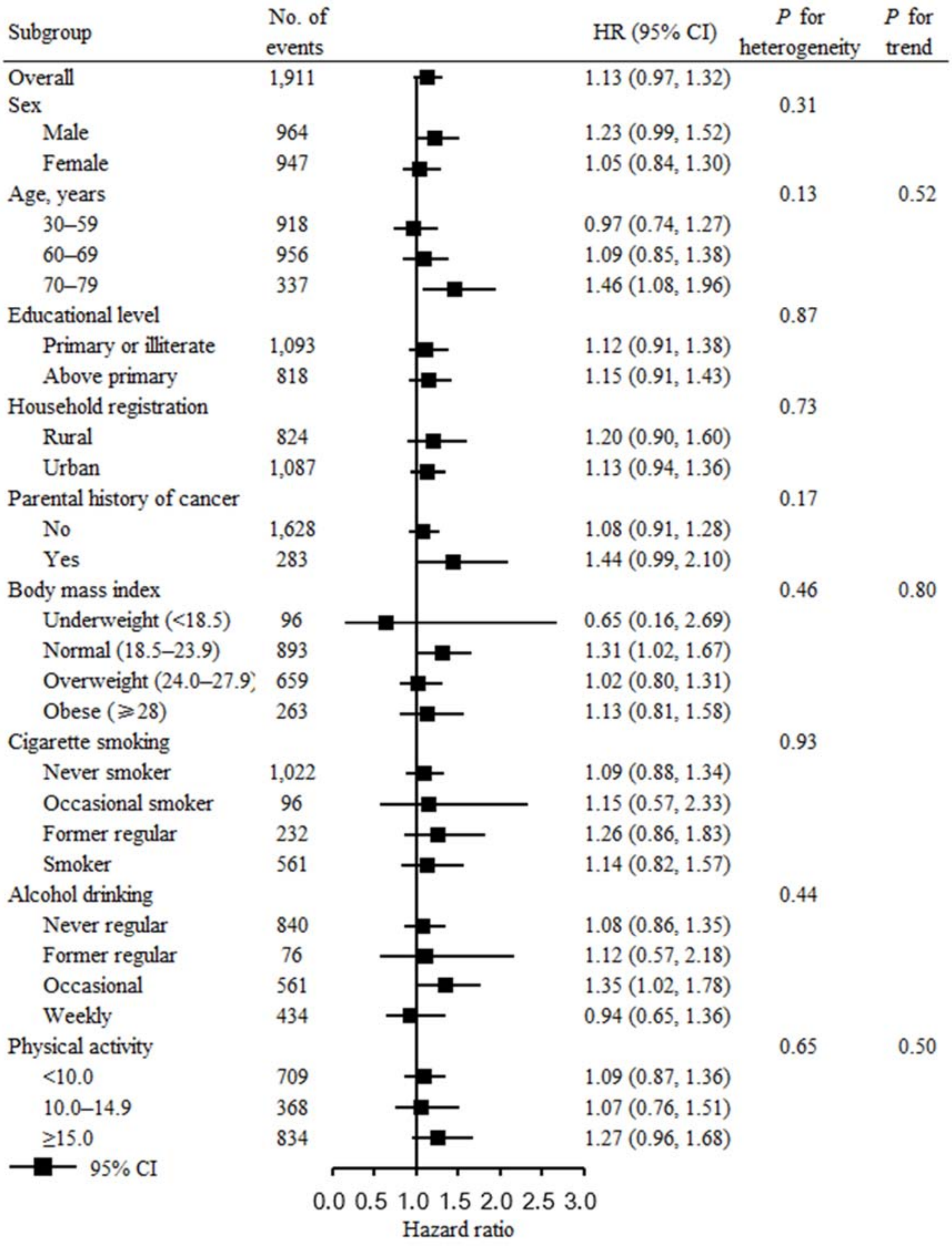

Web Figure 3. Adjusted hazard ratios (HRs) for colorectal cancer by type 2 diabetes, China Kadoorie Biobank Study, 2004-2013. Body mass index was defined as weight (kg)/height (m)^2; physical activity (PA) was estimated in terms of MET-hours/day spent on work, transportation, housework, and nonsedentary recreation. Bars, 95% confidence intervals (CIs). MET, metabolic equivalent of task.

Subgroup	No. of events	HR (95% CI)	*P* for heterogeneity	*P* for trend
Overall	3,527	1.11 (0.98, 1.25)		
Sex			0.71	
Male	2,154	1.13 (0.96, 1.32)		
Female	1,373	1.08 (0.91, 1.30)		
Age, years			0.25	0.43
30–59	1,492	1.25 (1.02, 1.53)		
60–69	1,360	0.99 (0.82, 1.19)		
70–79	675	1.13 (0.89, 1.44)		
Educational level			0.12	0.12
Primary or illiterate	2,249	1.19 (1.02, 1.39)		
Above primary	1,278	0.98 (0.81, 1.19)		
Household registration			0.29	
Rural	1,811	1.26 (1.03, 1.54)		
Urban	1,716	1.10 (0.95, 1.28)		
Parental history of cancer			0.99	
No	3,041	1.11 (0.97, 1.26)		
Yes	486	1.11 (0.81, 1.52)		
Body mass index			0.92	0.8
Underweight (<18.5)	291	1.32 (0.76, 2.28)		
Normal (18.5–23.9)	1,917	1.08 (0.90, 1.30)		
Overweight (24.0–27.9	1,041	1.08 (0.89, 1.31)		
Obese (≥28)	278	1.10 (0.80, 1.52)		
Cigarette smoking			0.83	
Never smoker	1,367	1.14 (0.95, 1.36)		
Occasional smoker	121	0.86 (0.41, 1.82)		
Former regular	439	1.20 (0.90, 1.60)		
Smoker	1,600	1.08 (0.88, 1.32)		
Alcohol drinking			0.42	
Never regular	1,497	1.20 (1.01, 1.42)		
Former regular	177	0.88 (0.52, 1.47)		
Occasional	909	0.96 (0.74, 1.24)		
Weekly	944	1.13 (0.88, 1.44)		
Physical activity			0.53	0.56
<10.0	1,365	1.12 (0.95, 1.32)		
10.0–14.9	707	1.22 (0.96, 1.57)		
≥15.0	1,455	1.00 (0.78, 1.27)		

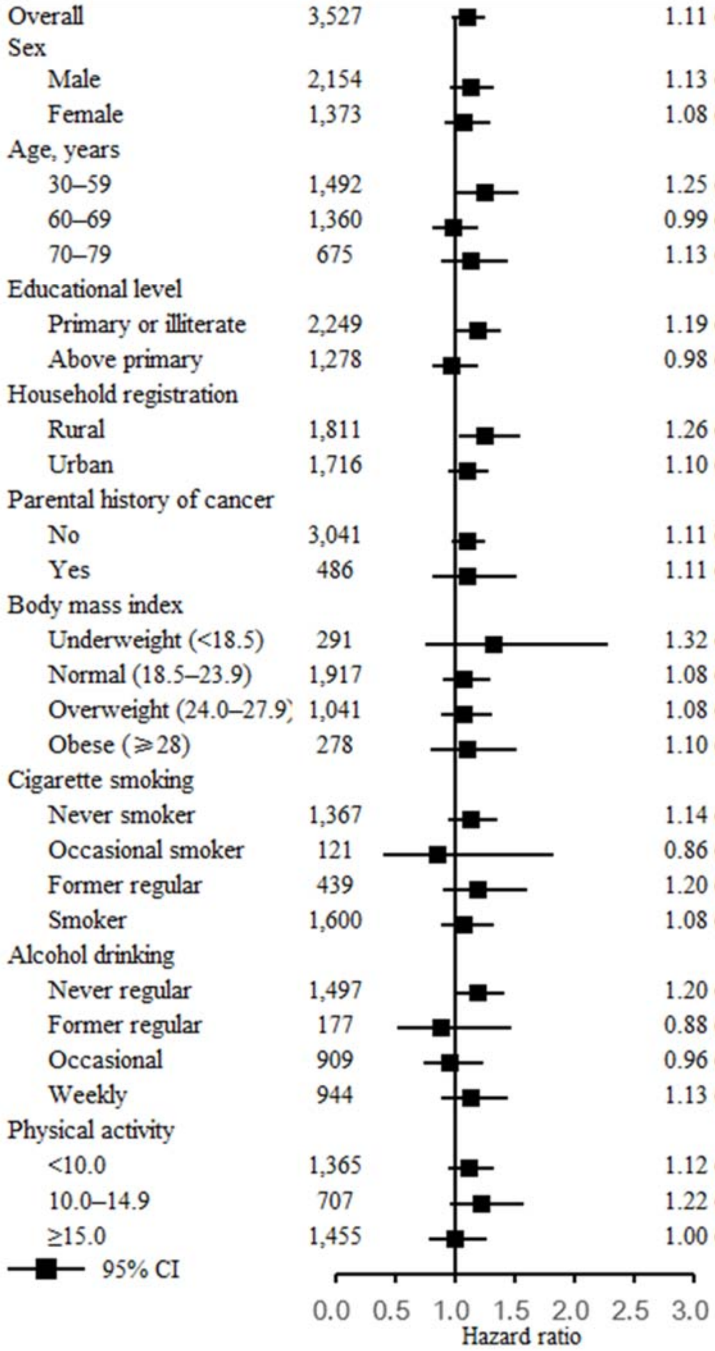

Web Figure 4. Adjusted hazard ratios (HRs) for lung cancer by type 2 diabetes, China Kadoorie Biobank Study, 2004-2013. Body mass index was defined as weight (kg)/height (m)^2; physical activity (PA) was estimated in terms of MET-hours/day spent on work, transportation, housework, and nonsedentary recreation. Bars, 95% confidence intervals (CIs). MET, metabolic equivalent of task.

Web Table 1. Association Between Type 2 Diabetes and Risk of Cancer, China Kadoorie Biobank Study, 2004-2013: Excluding Cancer Cases, Deaths, and Loss to Follow-up Occurring Within the First Three Years of Follow-Up [a]

Type of Cancer	No. of Events		HR [b]	95% CI
	T2DM	No T2DM		
All	873	10,206	1.10	1.02, 1.18
Esophagus	48	951	0.82	0.61, 1.11
Stomach	89	1,184	0.98	0.78, 1.22
Colon and rectum	122	1,142	1.11	0.92, 1.35
Liver	110	1,022	1.51	1.23, 1.85
Lung	168	2,084	0.96	0.82, 1.13
Female breast [c]	89	887	1.35	1.07, 1.69

Abbreviations: CI, confidence interval; HR, hazard ratio; T2DM, type 2 diabetes mellitus.

[a] 12,002 participants were excluded from the analysis, and the total sample size was 496,890. The follow-up for all participants started from three years after the baseline.

[b] Stratified by sex, age (5-year interval), and study area structure of the study population. Results were adjusted for education, parental history of cancer, body mass index, cigarette smoking, alcohol drinking, and physical activity.

[c] For women only (N=294,635). Stratified by age (5-year interval), and study area. Results were adjusted for education, parental history of cancer, menopause status, body mass index, cigarette smoking, alcohol drinking, and physical activity.

Web Table 2. Association Between Type 2 Diabetes and Risk of Incident Cancer, China Kadoorie Biobank Study, 2004-2013: Excluding Participants With Cardiovascular Disease and Other Major Chronic Disease [a]

Type of Cancer	No. of Events		HR [b]	95% CI
	T2DM	No T2DM		
All	1,200	14,629	1.14	1.08, 1.21
Esophagus	79	1,482	0.89	0.71, 1.13
Stomach	133	1,924	0.97	0.81, 1.15
Colon and rectum	156	1,568	1.16	0.98, 1.37
Liver	147	1,478	1.49	1.25, 1.77
Lung	250	2,905	1.12	0.98, 1.27
Female breast [c]	108	1,234	1.30	1.06, 1.59

Abbreviations: CI, confidence interval; HR, hazard ratio; T2DM, type 2 diabetes mellitus.

[a] 29,447 participants with major chronic diseases were excluded from the analysis, and the total sample size was 479,445.

[b] Stratified by sex, age (5-year interval), and study area structure of the study population. Results were adjusted for education, parental history of cancer, body mass index, cigarette smoking, alcohol drinking, and physical activity.

[c] For women only (N=284,189). Stratified by age (5-year interval), and study area, and adjusted for education, parental history of cancer, menopause status, body mass index, cigarette smoking, alcohol drinking, and physical activity.

Web Table 3. Association Between Type 2 Diabetes and Risk of Incident Cancer, China Kadoorie Biobank Study, 2004-2013: Additional Adjustment for Dietary Factors

Type of Cancer	T2DM versus no T2DM	
	HR [a]	95% CI
All	1.12	1.06, 1.19
Esophagus	0.85	0.68, 1.06
Stomach	0.89	0.75, 1.06
Colon and rectum	1.13	0.97, 1.32
Liver [b]	1.52	1.30, 1.77
Lung	1.11	0.98, 1.25
Female breast [c]	1.25	1.04, 1.51

Abbreviations: CI, confidence interval; HR, hazard ratio; T2DM, type 2 diabetes mellitus.

[a] Stratified by sex, age (5-year interval), and study area structure of the study population. Results were adjusted for education, parental history of cancer, body mass index, cigarette smoking, alcohol drinking, physical activity, meat consumption, consumption of fresh vegetables, and consumption of fruits.

[b] Stratified by sex, age (5-year interval), and study area structure of the study population. Results were adjusted for education, parental history of cancer, body mass index, cigarette smoking, alcohol drinking, physical activity, meat consumption, consumption of fresh vegetables, consumption of fruits, and hepatitis/cirrhosis.

[c] For women only (N=300,060). Stratified by age (5-year interval), and study area. Results were adjusted for education, parental history of cancer, menopause status, body mass index, cigarette smoking, alcohol drinking, physical activity, meat consumption, consumption of fresh vegetables, and consumption of fruits.

Web Table 4. Association Between Type 2 Diabetes and Risk of Cancer, China Kadoorie Biobank Study, 2004-2013: Treating Diagnosed Diabetes Cases as Time-varying During Follow-up

Type of Cancer	No. of Events		HR [a]	95% CI
	T2DM	No T2DM		
All	1,083	16,380	1.31	1.23, 1.40
Esophagus	47	1,612	0.84	0.63, 1.13
Stomach	104	2,105	1.04	0.85, 1.28
Colon and rectum	129	1,782	1.13	0.94, 1.35
Liver	143	1,797	1.75	1.47, 2.08
Lung	251	3,276	1.37	1.20, 1.56
Female breast [b]	88	1,434	1.24	0.99, 1.56

Abbreviations: CI, confidence interval; HR, hazard ratio; T2DM, type 2 diabetes mellitus.

[a] Stratified by sex, age (5-year interval), and study area structure of the study population. Results were adjusted for education, parental history of cancer, body mass index, cigarette smoking, alcohol drinking, and physical activity.

[b] For women only (N=294,165). Stratified by age (5-year interval), and study area. Results were adjusted for education, parental history of cancer, menopause status, body mass index, cigarette smoking, alcohol drinking, and physical activity.
